# Emerging and Re-Emerging Diseases Caused by Badnaviruses

**DOI:** 10.3390/pathogens12020245

**Published:** 2023-02-03

**Authors:** Alangar Ishwara Bhat, Ramasamy Selvarajan, Velusamy Balasubramanian

**Affiliations:** 1 Division of Crop Protection, ICAR-Indian Institute of Spices Research, Kozhikode 673012, Kerala, India; 2 Division of Crop Protection, ICAR-National Research Centre for Banana, Trichy 620102, Tamil Nadu, India

**Keywords:** pararetrovirus, reverse transcribing virus, endogenous virus, episomal virus, detection, management

## Abstract

New and emerging plant diseases are caused by different pathogens including viruses that often cause significant crop losses. Badnaviruses are pararetroviruses that contain a single molecule of ds DNA genome of 7 to 9 kb in size and infect a large number of economically important crops such as banana and plantains, black pepper, cacao, citrus, grapevine, pineapple, sugarcane, sweet potato, taro, and yam, causing significant yield losses. Many of the species in the genus have a restricted host range and several of them are known to infect a single crop. Combined infections of different virus species and strains offer conditions that favor the development of new strains via recombination, especially in vegetatively propagated crops. The primary spread of badnaviruses is through vegetative propagating materials while for the secondary spread, they depend on insects such as mealybugs and aphids. Disease emerges as a consequence of the interactions between host and pathogens under favorable environmental conditions. The viral genome of the pararetroviruses is known to be integrated into the chromosome of the host and a few plants with integrants when subjected to different kinds of abiotic stress will give rise to episomal forms of the virus and cause disease. Attempts have been made to develop management strategies for badnaviruses both conventionally and using precision breeding techniques such as genome editing. Until 2016 only 32 badnavirus species infecting different crops were known, but in a span of six years, this number has gone up to 68. The current review highlights the emerging disease problems and management options for badnaviruses infecting economically important crops.

## 1. Introduction 

Badnaviruses are plant pararetroviruses that belong to the family *Caulimoviridae* and contain 68 species demarcated based on the sequence identity in the conserved reverse transcriptase (RT)/ribonuclease H (RNase H) coding region [1,2]. The family *Caulimoviridae* consists of 11 genera including *Badnavirus*, namely, *Caulimovirus* (12 species), *Cavemovirus* (three species), *Dioscovirus* (one species), *Petuvirus* (one species), *Rosadnavirus* (one species), *Ruflodivirus* (one species), *Solendovirus* (two species), *Soymovirus* (one species), *Tungrovirus* (one species), and *Vaccinivirus* (one species) [2]. Of these, *Dioscovirus* and *Ruflodivirus* have no particle morphology while badnaviruses and tungrovirus have bacilliform particles and the rest of the viruses have isometric particles. Genomes of all pararetroviruses comprise a double-stranded DNA and they replicate through an RNA intermediate. However, in contrast to retroviruses, integration of the viral genome is not mandatory for the replication of pararetroviruses. Instead, they accumulate as minichromosomes in the host nucleus. Illegitimate and generally fragmented integration occurs once in every million years [3]. 

Particles of badnaviruses are approximately 30 nm × 120–150 nm in size and their genome consists of a circular, double-stranded DNA genome of 7.2–9.2 kb coding for a minimum of three open reading frames (ORFs) [4,5]. Replication of these viruses occurs via reverse transcription of a greater-than-genome length RNA that serves as a template both for the translation of viral proteins and for reverse transcription to replicate the genome [4,6]. Further, genomes of some badnaviruses integrate into the chromosomes of their hosts through illegitimate recombination, and these linear forms of integrants are known as endogenous badnaviruses [5,7,8]. A few of such endogenous badnaviruses were shown to give rise to the episomal form of the virus (upon exposure to abiotic stress) that cause systemic infection and spread from plant to plant through insect vectors [5,9,10,11]. 

Badnaviruses are one of the emerging groups of viruses infecting a large number of economically important crop plants, especially in the tropical and subtropical regions of the world [6]. The number of ICTV-recognized badnavirus species in 2016 was just 32 [5], while this number has now increased to 68 species in a span of just six years [2] (Table 1), indicating their importance. Combined infections of different virus species and strains offer conditions that favor the development of new strains via recombination, especially in vegetatively propagated crops. These viruses are reported to cause negligible to significant yield loss varying between 10 and 90% in different crops. 

**Figure 1:**
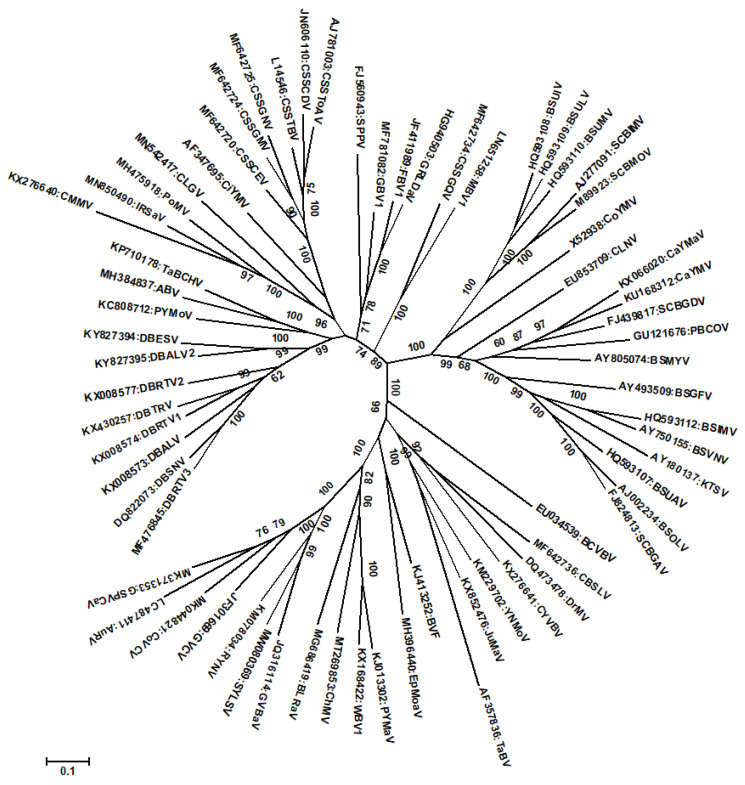
Phylogenetic tree constructed using full-length genome sequences of badnavirus approved by the ICTV. The evolutionary history was inferred by using the Maximum Likelihood method based on the JTT matrix-based model conducted in MEGA7 [17]. Bootstrap values for 1000 replicates are given when above 50%. The tree is drawn to scale, with branch lengths measured in the number of substitutions per site. The GenBank accession numbers and acronyms of viruses are shown while the details of acronyms used are provided in Table 1.

Although badnaviruses occur throughout the world both in monocots and dicots, they cause economically important diseases in tropical and subtropical regions on perennial crops such as banana, black pepper, citrus, cocoa, grapevine, pineapple, sugarcane, sweet potato, taro, and yam. Many of the species in the genus have a restricted host range and several species are known to infect a single crop (cocoa, banana, yam, sugarcane, and grapevine are infected by ten, nine, eight, four, and three distinct species respectively, while pineapple and taro are infected by two species each) (Table 1) (Figure 1). This kind of mixed infection of a single plant by different virus species and strains might lead to the development of new strains/species through recombination, especially in vegetatively propagated crops.

The viral genome of a few badnaviruses and other pararetroviruses is known to be integrated into the chromosome of the host through illegitimate recombination. These sequences are known as endogenous pararetroviruses (EPRVs) [7,12,13,14]. In most cases, EPRVs denote only partial, linear forms of the original circular viral DNA, do not cause any symptoms or disease in the plants, and may provide viral immunity [15]. Since there is sequence similarity between the endogenous and episomal virus sequence, PCR assay may not be able to differentiate them instead, methods such as Southern or in situ hybridization of the chromosome with the viral probe, immunocapture (IC)-PCR, rolling circle amplification (RCA), reverse transcription-PCR, electron microscopy, and host genome sequencing are needed to distinguish episomal and integrated forms of the virus. The integrants of three badnaviruses infecting bananas (banana streak OL virus, BSOLV; banana streak GF virus, BSGFV and banana streak IM virus, BSIMV) give rise to episomal forms in some hybrid plants under abiotic stress conditions such as temperature and tissue culture [9,11,13,16]. The episomal forms of these viruses can cause disease in plants and spread to other plants through mealybug vectors [5].

**Figure 2:**
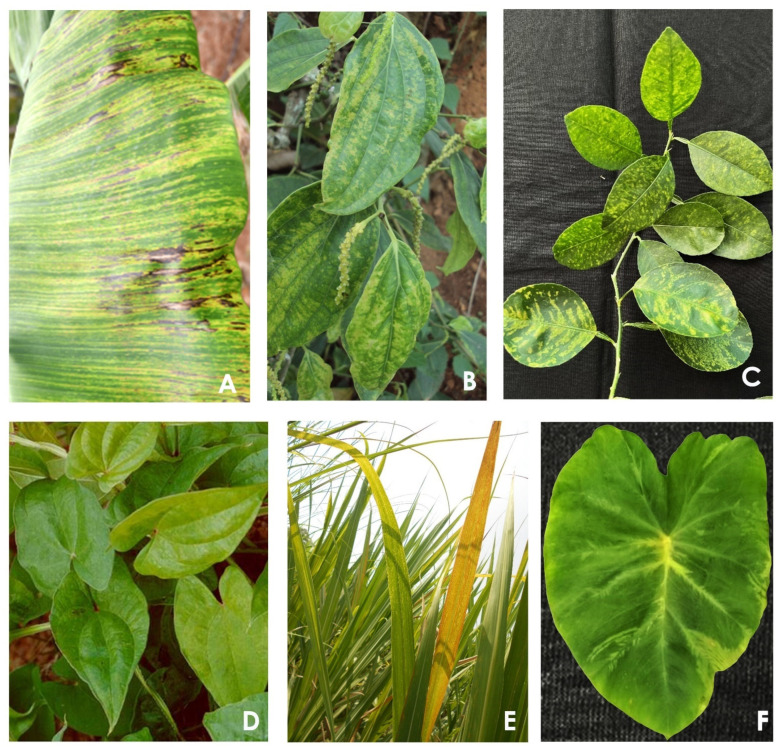
Symptoms caused by badnaviruses on different hosts: (**A**) banana streak virus on banana, (**B**) piper yellow mottle virus on black pepper, (**C**) citrus yellow mosaic virus on citrus (source: Dilip Ghosh, ICAR-Central Citrus Research Institute, Nagpur, India), (**D**) Dioscorea bacilliform virus on yam (source: Makeshkumar, ICAR-Central Tuber Crops Research Institute, Thiruvananthapuram, India), (**E**) sugarcane bacilliform virus on sugarcane (source: R. Viswanathan, ICAR-Indian Institute of Sugarcane Research, Lucknow, India), and (**F**) taro bacilliform virus on taro (source: T. Makeshkumar, ICAR-Central Tuber Crops Research Institute, Thiruvananthapuram, India).

Badnaviruses cause variable symptoms ranging from asymptomatic, mild, and moderate to severe in the hosts that are influenced by abiotic factors, especially environmental conditions and the nutrient status of the crop (Figure 2). Hence, symptoms are not reliable criteria for the detection of badnavirus infection on a plant. Serological assays are also not reliable due to the low titer and heterogeneity of virus isolates infecting a plant. PCR is the most commonly used assay for the detection of the virus. However, PCR may sometimes detect both endogenous and episomal viruses. Thus, to detect the presence of the episomal form of the virus, RT-PCR or rolling circle amplification (RCA) may be used.

## 2. Emerging Disease Problems in Plants

### 2.1. Banana

Banana streak virus disease was first reported in Morocco in the year 1974 by the Lockhart group from the University of Minnesota. Later it was reported to occur across the world including many countries in Africa, Asia, Central, and South America, and the Pacific. It is now known that wherever bananas are grown the streak virus also exists. Banana streak virus induces typical chlorotic or necrotic streak symptoms not only on the leaf lamina but also on the midrib, leaf sheaths, and peduncle. The chlorotic streaks are narrow, small, discontinuous, and parallel to the veins and subsequently, these streaks are turned into necrotic, sometimes splitting of the pseudostem is observed to be due to the virus infection. Internal necrosis of the pseudostem, abnormal bunch emergence by piercing through the pseudostem, and necrotic streaks on fruits have also been recorded. Peel splitting and necrotic spots on fruits are reported to occur. The symptoms are not uniformly distributed on all leaves of the infected tree, and the temperature plays a major role in the type and severity of the of symptoms. The virus titer and the symptoms keep changing due to the temperature and often there will be symptomless growth for a long period, and it reappears suddenly on a new leaf. At 22 °C plants tend to express the symptoms of streak conspicuously whereas above 28 °C to 35 °C, the symptoms are invisible and if the plants are moved to a growth chamber at 22 °C the symptoms are significantly increased and become severe [18,19]. Plants of Pome banana variety Virupakshi (AAB) when subjected to 22 °C for six months to a year are characterized by the expression of streak symptoms, and the virus associated was identified as BSGFV based on the PCR and sequencing of RCA amplified products. Daniells et al. [20] reported a yield reduction of 6% in Cavendish besides an 18 days delay in harvest under Australian conditions. Dallol et al. [9] reported that the process of micropropagation and the hybrids and interspecific natural hybrids of *Musa accuminata* and *M. balbisiana* express severe symptoms of streak upon tissue culturing and they assumed to be due to the expression of virus from the endogenous sequence.

**Figure 3:**
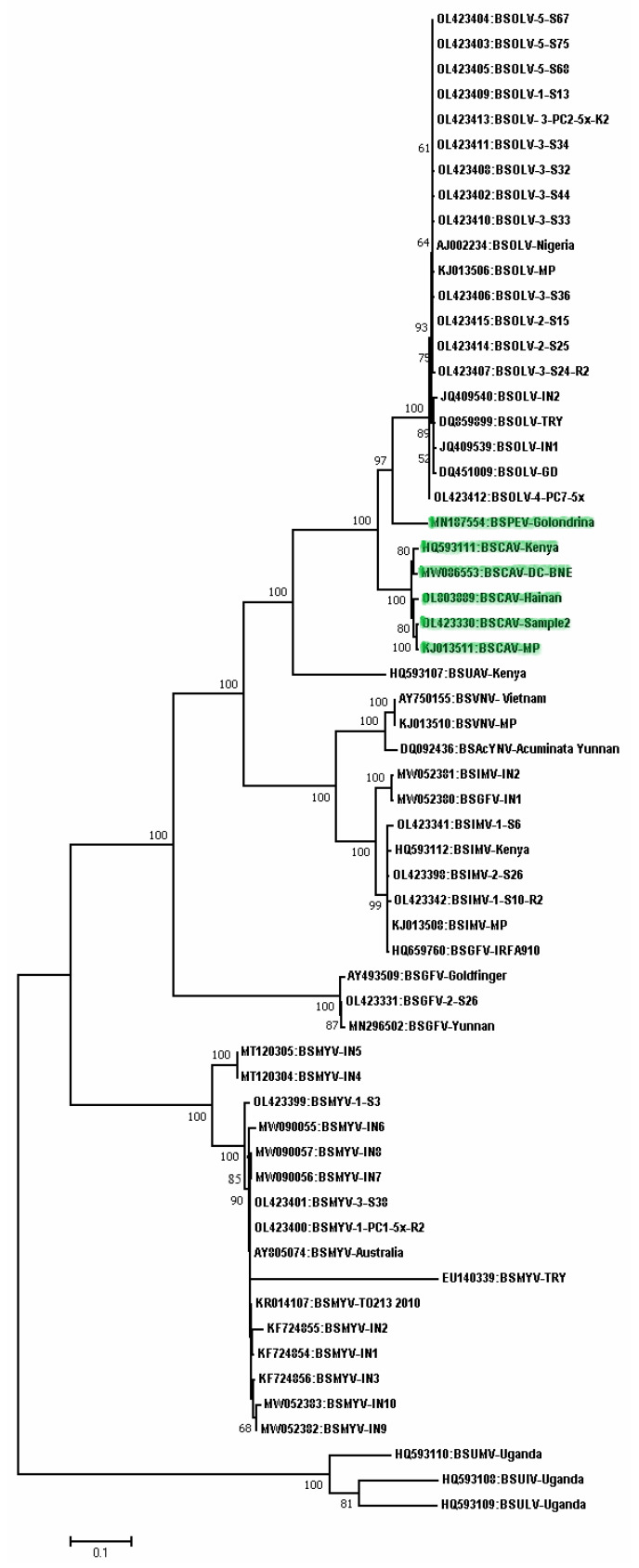
Phylogenetic tree constructed based on the amino acid sequences of open reading frame III from isolates belonging to different BSV species available in the GenBank. The evolutionary history was inferred by using the Maximum Likelihood method based on the JTT matrix-based model conducted in MEGA7 [17]. Bootstrap values for 1000 replicates are given when above 50%. The tree is drawn to scale, with branch lengths measured in the number of substitutions per site. The GenBank accession numbers and acronyms of viruses are shown while the details of acronyms used are provided in Table 1. The tentative species are highlighted in green.

BSVs are primarily transmitted through infected planting materials such as corms, corm bits, and infected plants mass propagated through tissue culture and the secondary transmission is through sap-feeding mealy bugs such as *Planococcus citri,* which transfers the virus in a semi-persistent manner. Lockhart et al. [21] reported BSV initially but later many distinct species of BSV infecting bananas were reported based on the sequence data of the whole genome or RT/RNaseH region. The very first isolate of BSV was later named BSOLV which was from Nigeria and the complete genome of this isolate had 7389 bp with three Open Reading Frames (ORFs) coding proteins of 20.8. 14.5 and 208 KDa of which the polyprotein contained putative movement protein, aspartic protease, RT/RNaseH, and coat protein. BSOLV showed high similarity with another badnavirus infecting sugarcane namely, sugarcane bacilliform virus (SCBV). Subsequently, Geering et al. [22,23] reported two additional new species from Australia and named them banana streak Mysore virus (BSMYV) and banana streak Gold finger virus (BSGFV); later, Harper et al. [24,25] described 13 new BSV species based on the extreme variability in RT/RNaseH sequences from Uganda. Further, Geering et al. [26] reported a complete genome sequence of the banana streak IM virus (BSIMV), and Lheureux et al. [27] reported two distinct complete genome sequences, namely banana streak VN virus (BSVNV) and banana streak Yunnan virus. Currently, nine BSV species are recognized by the International Committee on Taxonomy of Viruses (ICTV), namely, banana streak GF virus (BSGFV), banana streak IM virus (BSIMV), banana streak MY virus (BSMYV), banana streak OL virus (BSOLV), banana streak UA virus (BSUAV), banana streak UI virus (BSUIV), banana streak UL virus (BSULV), banana streak UM virus (BSUMV), and banana streak VN virus (BSVNV) (Figure 3). There are also other tentative species, such as banana streak CA virus (BSCAV), banana streak Peru virus (BSPEV), banana streak UJ virus (BSUJV), banana streak UK virus (BSUKV), and banana streak acuminata Yunnan virus (BSAcYNY) awaiting ICTV recognition (Figure 3).

In bananas, being a clonally propagated fruit crop, detection of viruses in the suckers or corms or corm bits is essential to ensure virus-free planting materials to the growers. Sensitive detection methods are the key to certifying the germplasm and mother plants used for mass propagation free of viruses. BSVs are serologically heterogeneous, and hence serological-based detection is cumbersome; however, IC-RT-PCR is applied using antiserum raised against a mixture of BSVs and SCMV [21]. Moreover, these pararetroviruses have their genomes integrated into the chromosomes, affecting the PCR-based detection of BSV as false positives can be possible from eBSVs. Bananas with a ‘B’ genome always have eBSVs whereas ‘A’ genome varieties are devoid of such integrants. Activatable eBSVs such as BSOLV, BSIMV, and BSGFV and episomal BSVs could be differentiated using RCA and PCR-based approaches, especially for banana plants that contain the ‘B’ genome, while for banana plants with ‘A’ genomes, RCA is the only option to distinguish between endogenous and free viral genomes [5]. Different species of BSVs (including the three species that harbour activable endogenous viruses) causing the streak disease were shown to belong to clade 1 of badnaviruses while clade 2 consisted only of endogenous BSVs that lack episomal counterparts [28]. Clade 3 consisted of species of BSVs from East African countries that also cause streak disease but lack endogenous counterparts. Based on the three abovementioned clades, a model for the co-evolution of virus and banana was proposed and it was suggested that endogenous badnaviral sequences can be used as markers to characterize the *Musa* phylogeny [28,29]. Recombination analysis of BSV and SCBV sequences detected a total of 32 recombinants between BSV and SCBV, besides many intra-BSV and intra-SCBV recombinants [30]. The breakpoints in the recombinants are mainly located in the intergenic region and C-terminal of ORF3 in both species of the viruses. The unique fragments detected in the analysis suggest the possibility that BSV and SCBV or their ancestors infected the same host before making the host shift.

A real-time PCR was reported for the rapid detection of episomal BSOLV and BSMYV [31], immunocapture PCR (IC-PCR), multiplex immunocapture PCR (M-IC-PCR), and rolling circle amplification (RCA) have been developed for the detection of episomal circular BSV genomes [32,33,34,35]. Reverse transcription PCR (RT-PCR) was successfully used to target the transcripts of episomal BSMYV [36,37]. James et al. [33] developed the RCA method using BSV-specific primers and demonstrated the ability of this method to differentiate between episomal and integrated viral genomic sequences. Serological-based specific detection was reported for BSMYV using the antibodies produced against the bacterially expressed virus-associated protein encoded by ORF II [38]. Zhang et al. [39] developed an RT-LAMP multiplex assay to detect three different DNA and RNA viruses in bananas. The reverse transcription-recombinase polymerase amplification assay (RT-RPA) has been developed for the detection of episomal BSMYV [40]. Recently, a rapid, sensitive, and simultaneous detection method was developed for BSV, BBTV, and CMV using a bead-based multiplex assay has been used for diagnosis [41].

Elimination of virus from the infected propagule using cryotherapy has become successful for many plant viruses. Helliot et al. [42] reported that 90% of virus elimination could be achieved in cv. Williams (AAA) of the Cavendish subgroup using cryopreservation of meristem through vitrification by plant vitrification solution 2 (PVS-2) solution whereas only 76% elimination was achieved by meristem tip culture. Cryopreservation followed by apical meristem culture has a high rate of success in eliminating episomal BSV infections in cv. Williams, a banana genotype free of eBSV [42]. Two anti-retroviral and anti-hepadnavirus molecules have also been effective in eradicating the episomal form of BSV from banana plants [43]. Mutating the eBSV sequences of bananas shall stop the spontaneous production of episomal virus and may stop the expression of the symptoms. Genome editing is widely used in agriculture to add new traits or to control viral pathogens by manipulating the host and virus protein interactions. Tripathi et al. [44] applied a CRISPR/Cas9-based genome-editing approach for banana and demonstrated by inactivation of the endogenous eBSV sequence integrated into the ‘B’ genome of plantain using multiple gRNAs.

### 2.2. Black Pepper

The yellow mottle disease (also known as stunt disease) of black pepper (*Piper nigrum*) caused by the badnavirus, *Piper* yellow mottle virus (PYMoV) is reported in Brazil, China, India, Indonesia, Malaysia, Philippines, Sri Lanka, Thailand, and Vietnam [45,46,47,48,49]. The disease is characterized by chlorotic flecks, mild to severe mottles, and brittle, leathery, deformed, and reduced leaf size (Figure 2). The length between the nodes of infected plants is reduced at the later stage, leading to the stunting of the entire vine, hence the name stunt disease [47]. The yield loss due to the disease may vary from negligible to up to 80% depending on the severity of the disease. Symptom expression and disease severity are positively correlated with high temperature and high relative humidity [50]. Severe diseases also develop under poor nutrition of the plants. Thus, soil and plant health play an important role in the symptom expression and the severity of the disease. Lockhart et al. [45] for the first time reported the association of a distinct badnavirus with yellow mottle disease affecting black pepper from Indonesia, Malaysia, Philippines, and Thailand and named it *Piper* yellow mottle virus (PYMoV) based on bacilliform virus particles, transmission by mealybug, its weak serological relationship with other badnaviruses, and its partial nucleotide sequence of the reverse transcriptase (RT)/RNase H region of the virus. Later association of PYMoV with black pepper was reported in Brazil [51], Sri Lanka [46], India [47], Vietnam, and China [48]. In addition to black pepper, PYMoV also infects other economically important *Piper* species such as *P. betle* (betelvine), *P. longum* (Indian long pepper), and many other wild *Piper* species [52,53]. The first complete genome sequence of the PYMoV of an Indian isolate was 7622 nucleotides long, potentially coding for four ORFs of proteins with 16, 17, 219, and 17 kDa, respectively [54]. Currently, the complete genome sequence of six isolates of PYMoV infecting black pepper, betelvine, and Indian long pepper originating from India and China showed that the length of the genome varied from 7557 to 7584 nucleotides and all isolates possessed four ORFs [48,55]. Of this, lengths of ORF 2 and ORF 4 showed country-specific characteristics. Further, the complete genome phylogenetic tree also showed country-specific clustering of PYMoV isolates [56]. The ORF III of the PYMoV codes a polyprotein with homology to different proteins such as viral movement, trimeric dUTPase, zinc finger, aspartic protease, reverse transcriptase, and RNase H, while proteins coded by the other three ORFs (I, II, and IV) do not show homology to any known proteins. PYMoV showed 39% to 56% identity with other badnaviruses and phylogenetic analysis using either the complete genome or conserved region of the RT/RNaseH showed grouping of all PYMoV isolates into a single cluster separated from other badnaviruses. Black pepper is a perennial and propagated by vegetative means; the primary spread of the virus occurs through vegetative means while secondary spread in the field happens through semi-persistent transmission by mealybugs (*Ferrisia virgata* and *Planococcus citri*) and black pepper lace bug [45,46,47]. Methods such as PCR, real-time PCR, loop-mediated isothermal amplification (LAMP), and recombinase polymerase amplification (RPA) are reported for the identification of virus-free mother plants for propagation [57,58,59,60]. Further, the elimination of PYMoV through somatic embryogenesis combined with pre-treatment with an antiviral agent, ribavirin @20 mg/mL, and meristem tip culture combined with chemotherapy was reported [61]. Production and use of virus-free planting materials should be the main focus for the management of the virus. Under field conditions, controlling the mealybug vector, correcting the soil acidity and NPK application (based on the soil test), spraying with a micronutrient mixture, and application of farm yard manure and plant growth-promoting rhizobacteria consortia are recommended [62].

### 2.3. Citrus

A badnavirus associated with the yellow mosaic disease in citrus from India was reported based on the morphology of virus particles, serological reaction, and the partial genome sequence by Ahlawat et al. [63], and the causative virus was named citrus yellow mosaic virus (CYMV). The typical symptoms of the disease are vein flecking and yellow mosaic on the leaves of sweet oranges with an incidence as high as 10–70% and a yield loss of 77% with lesser juice and ascorbic acid content. Dodder is known to transmit CYMV to different citrus species such as sweet and sour oranges, rangpur lime, Pummelo, and Volkamer lemon. Interestingly the CYMV is also transmitted by mechanical sap inoculation to sweet orange, pummelo, and *Citrus decumana*. As with all the other badnaviruses, CYMV is also a non-enveloped bacilliform virus having a dimension of 150 × 30 nm in size with a 7559 bp length circular dsDNA with some discontinuous in its genome [63,64]. An infectious clone of CYMV was shown to infect sweet oranges through agro-infection [64]. Vadlamudi et al. [65] reported that ORFI of CYMV possesses RNA-silencing suppression activity. The complete genome sequence of CYMV from sweet orange, rangpur lime, acid lime, and pummelo shared >90% identity [66,67]. Endogenous florendovirus has also been reported to occur in citrus [68]. An in vitro-expressed virion-associated protein of the virus was reported to be useful for the detection of the virus [69]. Dot blot hybridization, RT-PCR, real-time PCR, and RPA-based methods have been reported for the detection of the virus in plants [69,70,71,72].

### 2.4. Cacao

Cacao swollen shoot disease (CSSD) of cacao (*Theobroma cacao* L.) was first discovered in Ghana in 1936 and later in other West African countries such as Ivory Coast, Liberia, Nigeria, Senegal, Sierra Leone, and Togo [73]. It is also reported in other countries such as Brazil [74], Indonesia [75], Sri Lanka [76], and Trinidad [77,78]. The typical symptom of the disease is a swollen shoot, which may be rounded on the end, and necrosis and death [79]. The foliar symptoms include vein-clearing, mosaic/mottling, red vein-banding, fern-like patterns, and leaf necrosis [80]. Both virulent and mild strains of the virus are known to occur with severe strains, causing the rapid decline and sudden death of plants [81]. The diseased plants produce a few smaller and discolored pods with poor bean quality. Agro-inoculation of the viral genome into the cacao plant showed the presence of virions in the cytoplasm of phloem companion cells and a few xylem parenchyma cells [82]. Further, stem swelling occurs due to the proliferation of the xylem, phloem, and cortex cells. The CSSD-associated viruses are transmitted semi-persistently by *Planococcoides njalensis* and *Planococus citri* and at least other 12 species of mealybugs and also through seeds and grafting [83,84,85].

Initially, the disease was thought to be caused by different strains of the virus that cause mild, moderate, and severe symptoms. Later, viral genomic sequence data indicated that many distinct badnavirus species are associated with the disease. Currently, ICTV lists ten different cacao swollen shoot virus species associated with the disease in different cacao-growing regions. They include cacao bacilliform Sri Lanka virus (CBSLV), cacao mild mosaic virus (CaMMV), cacao swollen shoot CD virus (CSSCDV), cacao swollen shoot CE virus (CSSCEV), cacao swollen shoot Ghana M virus (CSSGMV), cacao swollen shoot Ghana N virus (CSSGNV), cacao swollen shoot Ghana Q virus (CSSGQV), cacao swollen shoot Togo A virus (CSSTAV), cacao swollen shoot Togo B virus CSSTBV, and cacao yellow vein banding virus (CYVBV) [2,86]. The different species associated with CSSD encode four to six ORFs named ORFs1-4, X, and Y and an intergenic region [87]. Of these, ORF 2 encodes a protein of about 14–15 kDa with homology to nucleic acid binding activity while ORF 3 encodes a polyprotein of about 210 kDa containing domains for the movement protein, coat protein, aspartic protease, and reverse transcriptase (RT) and ribonuclease H (RNaseH) proteins [74,75,77,78,86,87,88,89,90,91,92]. The functions of the remaining ORFs (ORF I, X, and Y) are unknown. Integrated badnaviral sequences in the genome of many cacao genotypes named endogenous *T. cacao* bacilliform virus (eTcBV) were reported [93]. Mixed infections, sequence variation, and recombination resulted in the evolution of new badnavirus species in cacao. Among various badnaviruses, CSSTBV showed the least nucleotide diversity while CSSCEV exhibited the highest variability [94]. Recombination analysis of 82 badnaviral genomes showed eight recombination events located between the intergenic region and ORF 2 in the West African species CSSTBV, CSSCDV, and CSSTAV. Similarly, a strain of the CaMMV (CaMMV-PR3) was identified as a possible recombinant, with CaMMV-BR as the major parent [94].

Serological and PCR amplification assays, though developed, failed to detect the virus in all symptomatic plants owing to the association of many badnaviral species in the disease. In a study using 189 samples, the detection potential of the primers varied from 4% to 23% with the four best primers [95] indicating the need for new approaches for the detection and diagnosis of different badnaviruses. Based on PCR using primers specific for different viruses, it was inferred that CSSTBV is the predominant species associated with diseased cacao in Côte d’Ivoire [74]. Management of the disease mainly focused on the eradication of virus-infected plants, resistance breeding, mild strain cross-protection, and control of mealybug vectors. Rogueing infected trees and replanting with tolerant genotypes showed only limited success in the management of the disease. Germplasms introduced from South America to west Africa were also susceptible to the disease. The mild strain CSSTBV-N1 protected the plants against the severe strain of the virus for 11–14 years thereafter additional field inoculation with the mild strain was needed for achieving continuous protection against the severe strain [96]. Barrier cropping is another effective way to reduce the disease spread while providing shade to virus-infected plants reduces symptom severity [97]. Production of virus-free cacao plants by eliminating the virus from the virus-infected plants through somatic embryogenesis was reported [98].

### 2.5. Dioscorea

Dioscorea bacilliform virus (DBV) infects several species of yam and negatively impacts production in all the regions of the world where the crop is grown. The common symptoms are chlorosis on veins and necrosis, and leaf distortions such as crinkling and puckering have been noticed. DBVs are primarily spread through vegetatively propagated tubers of yam which helps the virus to perpetuate, and it is also transmitted mechanically through the sap; secondarily, they are transmitted by multiple species of mealybugs of the family Pseudococcidae. Turaki et al. [99] reported that there are several species of badnaviruses infecting the germplasm of yam in West Africa. Besides the episomal virus, a diverse range of endogenous sequences of DBV (eDBV) has been reported to occur on the yams cultivated around the world and more particularly in West Africa. However, so far, no eDBVs are reported to form into episomal viruses through homologous recombination as reported in bananas.

**Figure 4:**
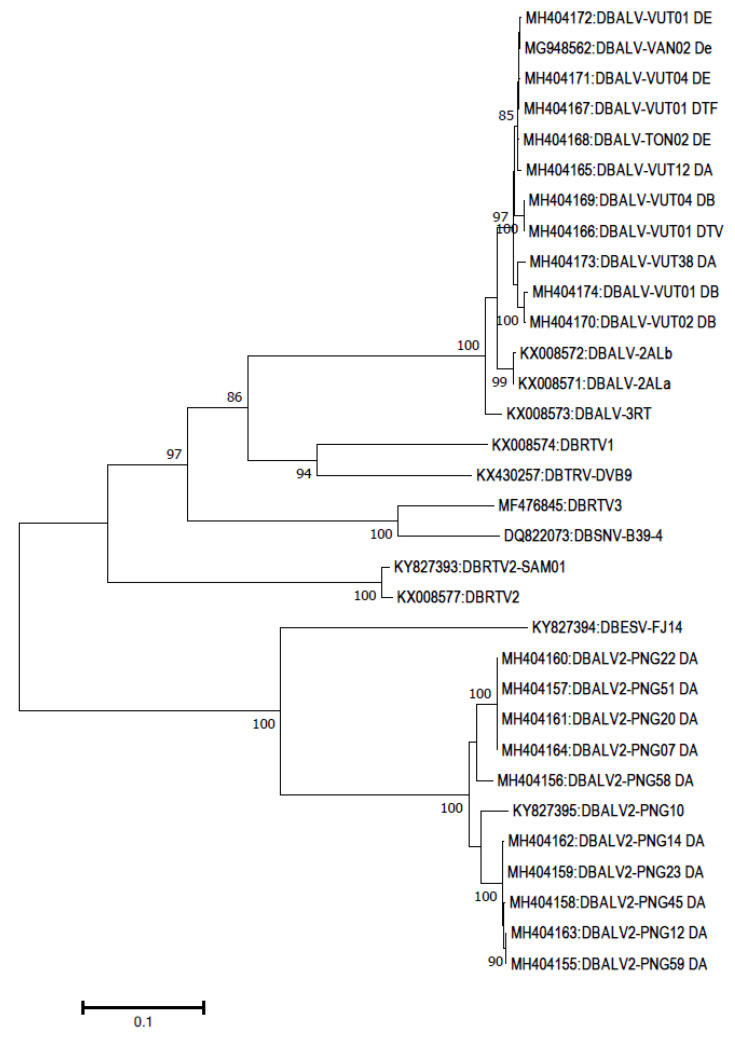
The phylogenetic tree was constructed based on the amino acid sequences of open reading frame III from isolates belonging to different dioscorea bacilliform virus species available in the GenBank. The evolutionary history was inferred by using the Maximum Likelihood method based on the JTT matrix-based model conducted in MEGA7 [17]. Bootstrap values for 1000 replicates are given when above 50%. The tree is drawn to scale, with branch lengths measured in the number of substitutions per site. The GenBank accession numbers and acronyms of viruses are shown while the details of acronyms used are provided in Table 1.

Both DBVs and eDBVs have been characterized by genome sequencing. The first complete genome sequence of DBV named DBALV is from Nigeria and infects *Dioscorea alata*. The complete genome sequence of two isolates of DBALV had 7413 and 7415 nt with three potential ORFs coding 29, 25, and 228 KDa proteins. Subsequently, two more isolates from Benin with 7262 and 7276 nt that showed 62% similarity with DBALV were named *Dioscorea sansibarensis* bacilliform virus (DBSNV). Later, another six distinct DBV genomes classified to be different species were completely sequenced; the six DBVs are, viz., dioscorea bacilliform AL virus 2 (DBALV2), dioscorea bacilliform ES virus (DBESV), dioscorea bacilliform RT virus 1 (DBRTV1), dioscorea bacilliform RT virus 2 (DBRTV2), dioscorea bacilliform RT virus 3 (DBRTV3) and dioscorea bacilliform TR virus (DBTRV) [100,101,102,103]. All the above-mentioned eight species are recognized by ICTV while seven more isolates are awaiting recognition [104] (Figure 4). The yam germplasm of the Pacific region contained four species (DBALV, DBALV2, DBRTV2, and DBRTV2). In Vanuatu and Tonga, DBALV was the most common species found whereas DBALV2, DBESV, and DBRTV2 were present in Papua New Guinea, Fiji, and Samoa. Recombination analysis of different DBV genomes revealed the occurrence of a unique recombination event located between the intergenic region and 5′ end of ORF 1 in the DBRTV3 with DBSNV and DBALV as major and minor parents, respectively [104].

DBV genomes are known to be integrated into the genomes of yam, which complicates the detection of the virus [102,105]. High heterogeneity among the episomal DBVs and lack of specific polyclonal and monoclonal antisera also hamper the detection of DBV by serological methods. Bomer et al. [100] developed an effective RCA protocol to identify, detect, and characterize episomal DBVs infecting yam. Turaki et al. [99] developed a PCR-dependent denaturing gradient gel electrophoresis (PCR-DGGE) for a quick and effective determination of complex mixtures of potentially episomal and endogenous DBV sequences. Onsite diagnostics using nanopore-based high-throughput sequencing for the discovery and reliable detection and sequencing of full-length genomes of yam viruses was reported [106]. The use of virus-free clean planting material, surveillance, vector management, and removal of alternate hosts of DBVs need to be integrated to manage the disease in the field.

### 2.6. Grapevine

Grapevine is infected by three badnaviruses of which the grapevine vein-clearing virus (GVCV) is one of the most important emerging viruses whose distribution is, so far, limited to the USA. GVCV causes symptoms such as vein-clearing of leaves, reduced internode length, and vine decline syndrome and it is transmissible by the aphid *Aphis illinoisensis* [107,108]. Grapevine Roditis leaf discoloration-associated virus (GRLDaV) causes yellow and reddish discoloration, deformity, and reduction in the leaf size and sugar content of the berries of grapevine, cv. “Roditis” in Greece, Italy, South Africa, and Turkey [109,110,111,112,113,114], while grapevine badnavirus 1 (GBV-1) is reported from Croatia [115]. Besides grafting, both GRLDDaV and GBV-1 are transmissible through the vine mealybug (*Planococcus ficus*) [114,115,116]. The complete genome of GVCV and GBV-1 contained 7753 and 7145 nucleotides, respectively, potentially coding for three typical ORFs while that of GRLDaV contained 6988–7090 nucleotides with four ORFs.

### 2.7. Pineapple

Virions of pineapple bacilliform virus (PBV) were first detected in pineapples [117], and later occurrence of a badnavirus-like sequence was detected in a large number of pineapple plants [118]. A degenerate PCR and immune-capture PCR were used to determine the diversity of badnaviruses in pineapples grown in Australia that indicated the occurrence of two new badnaviruses, the pineapple bacilliform comosus virus (PBCOV) and pineapple bacilliform erectifolius virus (PBERV), and an endogenous badnavirus, Endogenous pineapple pararetrovirus-1 (ePPPV-1), and a retrotransposon, *Ananas* metavirus (AMtV) [119]. Hawaiian pineapples also showed an occurrence of badnaviral sequences and were designated as pineapple bacilliform comosus virus-HI1 (PBCOV-HI1) along with nine genomic variants (A to H) [120]. The RT and RNaseH regions of PBCoV-HI1 showed 98% nucleotide similarity with PBCOV from China and Australia, suggesting that they are strains of a single species [121], whereas the amino acid level and the identity for ORF1, ORF2, and ORF3 were only 47% to 80%. The complete genome sequences of PBCOV isolates from China and Hawaii comprised 7543 and 7451 bp with the three ORFs [120,121]. Among the two, PBCOV is transmitted by mealybugs (*Dysmicoccus neobrevipes, D. neobrevipes,* and *Planococcus citri*) [117,119,120].

### 2.8. Sugarcane

The first recorded report of sugarcane bacilliform virus **(**SCBV) was from Cuba in 1985 followed by many other sugarcane-growing countries of the world through germplasm exchange due to lack of characteristic symptoms and vegetative propagation of the sugarcane [122,123]. Though the disease is characterized by symptoms such as chlorosis, mottle, and leaf freckle, many of the infected plants remain asymptomatic. However, symptoms are prominent when there is a mixed infection of SCBV with other viruses such as the sugarcane mosaic virus and sugarcane mild mosaic virus [123]. In China, SCBV-infected sugarcane is reported to decrease juice sucrose content, gravity purity, and stalk weight [124]. It is suggested that SCBV originated at the center of sugarcane origin in Papua New Guinea and spread to other countries. The secondary spread of the virus occurs semipersistently through mealybug vectors such as *Sacharicoccus sachhari* and *Dysmicoccus boninsis* [125,126,127]. Recently, true seed transmission of SCBV was shown in 18 crosses and their parents through PCR assays in India [128]. Even the pluff samples of parents used in the hybridization also tested positive for the virus, suggesting that the seed acquires the virus from the parents. Initial studies showed that SCBV and BSV were indistinguishable as SCBV-transmitted bananas showed symptoms similar to those described for BSV. Similarly, a Moroccan isolate of BSV and SCBV were serologically indistinguishable and a few isolates of BSV and SCBV could be transmitted mechanically to healthy sugarcane and banana, respectively [125]. Unlike many badnaviruses, SCBV has a relatively broad host range infecting members of the Poaceae and Musaceae, *Chenopdoium quinoa,* and tobacco [6]. SCBV is reported from noble canes, hybrids, and clones within the ‘Saccharum complex’ including *S. robustum*, *S. spontaneum*, *S. barberi*, *Erianthus arundinaceus,* and *E. ravennae* [129].

Currently, ICTV recognizes four species, namely, sugarcane bacilliform Guadeloupe A virus (SCBGAV), sugarcane bacilliform Guadeloupe D virus (SCBGDV), sugarcane bacilliform IM virus (SCBIMV), and sugarcane bacilliform MO virus (SCBMOV) while other isolates are still considered only as tentative species. Of these, the SCBMOV was the first to be completely sequenced, followed by SCBIMV [127,130]. The complete genome of the SCBMOV from Morocco contained 7568 nucleotides coding for typical three ORFs and an intergenic region [127]. Infectious clones of this virus when inoculated through agro-inoculation caused an infection in rice and banana plants. Complete genome sequencing of the SCBIMV from Australia (Ireng Maleng isolate) contained 7687 nucleotides that shared only 72% nucleotide sequence identity with SCBMOV. Later, based on the high sequence variability in the RT/RNaseH region among 35 isolates from Guadeloupe, seven phylogenic groups (A to G) including SCBMOV (group E) and SCBIMV (group F) were reported [131]. Based on the complete genome of the isolates from groups A and D, two more new species, namely, sugarcane bacilliform Guadeloupe A virus (SCBGAV) and sugarcane bacilliform Guadeloupe D virus (SCBGDV) were recognized by ICTV [131]. Similarly, Karuppaiah et al. [132] reported sequence variability ranging from 15 to 31% in the complete genome of five isolates of SCBV from India that showed 70–82% identity with SCBIMV and SCBMOV and suggested the creation of three more new species for which the names sugarcane bacilliform black Reunion virus (SCBBRV), sugarcane bacilliform BO virus (SCBBOV), and sugarcane bacilliform BB virus (SCBBBV) were proposed, which are yet to attain ICTV recognition. The complete genome sequence of two divergent strains of SCBV infecting sugarcane in China that aligned with SCBV-H and SCBV-G clades was reported [133]. Similarly, based on the RT/RNaseH region sequence analysis of 63 SCBV-infected sugarcanes from different regions of China, they were grouped into nine SCBV phylogroups of which phylogroups SCBV-S and SCBV-T were newly proposed [124]. Later, Janiga et al. [134] added five more phylogroups (SCBV-U to SCBV-Y) based on the RT/RNaseH region sequencing of 104 SCBV-infected sugarcane germplasms and *Saccharum* hybrids from India. Recently the occurrence of SCBV and its variability was reported in Ethiopia [135]. High genetic variability observed among SCBV isolates infecting sugarcane around the world clearly indicates the co-evolution of the virus and its host. Until now, complete genome sequences of 12 isolates of SCBV are available in the public domain. The occurrence of recombinant strains within SCBV and between SCBV and BSV strains was reported [133,136]. The putative species SCBV-U, SCBV-W, and SCBV-X and six other species were found to be recombinants, indicating the genetic exchange occurring between SCBV species over time, leading to the evolution of new strains/species [134]. The intergenic region was considered to be the hotspot for recombination followed by ORF1. Geijskes et al. [137] reported non-integration of the SCBV genome in the sugarcane chromosomes; however, based on PCR amplification and Southern hybridization, Cai et al. [138] reported integration of SCBV DNA fragments into the chromosomes of *Saccharum* inter-specific hybrids grown in China.

The variable region from SCBV was shown to serve as a promoter for high-level expression of foreign genes in both monocot and dicot transgenic plants [139,140]. miRNAs for silencing of the SCBV genome using in silico algorithms identified 14 potential candidate miRNAs from sugarcane, of which sof-miR159e was the top candidate that is capable of targeting ORF 3 of SCBV that can be exploited for the development of SCBV-resistant sugarcane plants [141].

### 2.9. Sweet Potato

Sweet potato pakakuy virus (SPPV) is a collective name coined for two distinct badnaviruses, namely, sweet potato badnavirus a (SPVa) and sweet potato badnavirus b (SPVb) obtained through siRNA sequencing of a virus-infected sweet potato landrace from Peru [142]. The complete genome of SPVa contained 8082 nt while that of SPVb contained 7961 nt, and both had four ORFs. The occurrence of both these viruses was also reported in Honduras, Guatemala, and Tanzania through small-RNA sequencing of symptomatic sweet potato plants [143,144]. The occurrence of an additional virus species based on the partial sequence of 3065 nt encoding putative movement and structural coat proteins, showing 86.3% and 73.1% identity to SPVb and SPVa, respectively, was also reported. PCR-based diagnostics and virus elimination through heat treatment combined with meristem tip culture were reported in Ethiopia for the management of the virus [145]. SPPV was omnipresent in very low titers in sweet potato germplasm and cultivars grown throughout the world and it does not occur in endogenous form. It is transmissible through seeds and cuttings and does not induce apparent symptoms [146]. Considerable variation was observed in the nucleotide sequences among isolates of the virus and it showed only a limited synergistic increase in the titers in plants infected with other viruses. Based on this it was concluded that these viruses persist and may not be a production constraint in sweet potatoes.

### 2.10. Taro

Taro (*Colocasia esculenta*) is a perennial aroid crop grown for its edible corms in Southeast Asia and the Pacific Island countries is infected by two badnaviruses, namely, taro bacilliform virus (TaBV) and taro bacilliform CH virus (TaBCHV) [2]. The disease is characterized by vein clearing, stunting, and downward curling of leaf blades, though sometimes infected plants do not show any external symptoms. TaBV and TaBCHV are transmitted by vegetation, seeds or pollen, and mealybugs (*Pseudococcus solomonensis*) [147,148]. TaBV and TaBCHV differ in their genome sequence and organization-TaBV containing four ORFs while TaBCHV contained six ORFs. Currently, the complete genome sequence of six isolates of TaBV representing Papua New Guinea, Australia, and East Africa and eight isolates of TaBCHV representing China, East Africa, and Hawaii are available. The complete nucleotide sequence of TaBV varied from 7458 to 7805 nucleotides in different geographical isolates with genome organization of ORFs I to III similar to the typical badnaviruses, while the ORFs IV and X are similar to those of ORF IV of CYMV and ORF X of CSSV and in the phylogenetic analysis, TaBV showed a close relationship with DBV [148]. Analysis of the coding region sequence of RT/RNaseH of different isolates representing different geographical regions also showed variability up to 23% and 33% in the coat protein region [149]. TaBCHV was first identified based on small-RNA sequencing of symptomatic taro plants from China, the complete genome of which showed 7641 nucleotides (identity with TaBV was only 55.8%) with the potential to code for six ORFs [150]. Currently, the complete genome of eight isolates of TaBCHV ranging in nucleotides from 7389 to 7647 with six ORFs encoding proteins of 16.8, 14.1, 206.4, 13.2, 11.9 and 12.4 kD is reported [2]. Yang et al. [151] reported a PCR assay for the detection of TaBV infecting taro and also suggested the possibility of the integration of TaBV sequences in the taro chromosome. A region of TaBV genome was successfully used as a promoter for the expression of foreign genes in bananas and tobacco, suggesting the possibility of using the same in plant expression vectors for high-level constitutive expression of transgenes both in dicotyledonous or monocotyledonous plants. The approaches suggested for the management include adhering to the international guideline for the taro movement including screening for viruses and developing virus-free healthy taro for farmers [152,153], besides the removal of infected plants and insecticide spray to control the vectors.

### 2.11. Badnaviruses Infecting Other Plants

Badnaviruses are also reported to infect several ornamentals, medicinal herbs, fruits, trees, and weeds [154,155,156,157,158,159,160,161,162,163,164,165,166,167,168,169,170,171,172,173,174,175,176,177,178,179,180,181,182,183,184,185,186,187,188,189]. Birch leaf roll-associated virus, pagoda yellow mosaic-associated virus, and mulberry badnavirus 1 are known to infect different tree crops (Table 1) [156,157,158]. Similarly, aglaonema bacilliform virus, aucuba ringspot virus, bougainvillea spectabilis chlorotic vein-banding virus, camellia lemon glow virus, canna yellow mottle-associated virus, canna yellow mottle virus, codonopsis vein-clearing virus, epiphyllum mottle-associated virus, ivy ringspot-associated virus, kalanchoë top-spotting virus, spiraea yellow leaf spot virus, wisteria badnavirus 1, and rubus yellow net virus are reported to infect different ornamentals (Table 1) [154,159,160,161,162,163,164,165,166,167,168,170,171,172,174,175,176], while cycad leaf necrosis virus infects the gymnosperm *Zamia fischeri* [177]. Viruses such as blackberry virus F, chestnut mosaic virus, fig badnavirus 1, gooseberry vein banding-associated virus, and jujube mosaic-associated virus infect different fruit crops [155,178,179,180,181,182,183,184,185], while polyscias mosaic virus and green Sichuan pepper vein clearing-associated virus infect medicinal and spice crops [186,187]. The yacon necrotic mottle virus infects the tuber crop, *Smallanthus sonchifolius* while commelina yellow mottle virus, the type species of the genus, *Badnavirus*, infects the weed plant (*Commelina diffusa*) [188,189]. Of these, yield loss data are not available for many viruses except for the rubus yellow net virus, which infects red and blackberry in North America and Europe, causing a loss of 30–75% in the first year in mixed infection with black raspberry necrosis virus [154]. The majority of these viruses cause vein clearing, mosaic, mottling, chlorosis, necrosis, leaf malformation, and stunting, and are transmitted by vegetative propagules, seeds, aphids, and mealybugs.

## 3. Recombination among Badnaviruses

Recombination analysis provides important information on the evolution of viruses. It is considered a major factor contributing to the genomic variability and diversity among badnaviruses. We subjected complete genome sequences of 215 badnaviruses available in the GenBank for recombination analysis using RDP, Geneconv, Boot-scan, Maximum Chi Square, Chimaera, SisterScan, and 3Seq implemented in the Recombination Detection Program (RDP) v.4 with default settings [190]. The statistical significance was inferred by a *p*-value lower than a Bonferroni-corrected cut-off of 0.05. Only recombination events detected by five or more of the different methods were considered to yield a statistically reliable prediction. Recombination events were considered credible when an event was identified by at least three detection methods with an associated *p* value < 0.05. The present analysis detected a total of 34 independent recombination events, the majority of which are located between the intergenic region and ORF 2 (Table S1). Of these, recombinants such as BSMYV-TRY, BSMYV-IN2, and BSMYV-IN9 were detected using seven RDP detection methods, followed by recombinants such as TaBV-Ke52, CYMV-ROL, and CYMV-Nagri, detected using six RDP detection methods, and recombinants such as JuMaV-HZ/AKS-6, CSSCDV-CI152-09, TaBCHV-PNG-K, BLRaV-BpenGer407526, detected using five RDP detection methods (Table S1). Among all, maximum recombinants were detected in the BSMYV-TRY isolate with five different break vent points followed by BSMYV-IN9 isolate with four predicted recombinants. For different recombinants of BSV species (except for the BSGF-Yunnan), either isolates of the same or different BSV species served as major and minor parents. For BSGF-Yunnan, BSOLV-5-S68 acted as the major parent, and SCBGAV1 as the minor parent. Similarly, for different recombinants of CYMV, in addition to CYMV isolates, an isolate of CSSGNV served as the major parent (Table S1). For recombinants JuMaV, in addition to isolates of JuMaV, CSSCDV, and PYMoV served as parents. Similarly, the four predicted recombinants of TaBV, in addition to TaBV, JuMaV, BSOLV, BSUMV, and ComYMV served as parents. For TaBCHV, DBALV2, YNMoV, CSSCEV, GRLDAV, DBALV2, and CYVBV served as major and minor parents. Some of the recombinant events in the present study were detected using unknown parents, suggesting the existence of undetermined diversity in badnaviruses. Mixed infection of a crop with more than one virus isolate/species might lead to the emergence of a new variant/species. The majority of badnaviruses do not cause any visible external symptoms; cultivation of such plants may facilitate the transmission of viruses between cultivars/varieties/germplasm, leading to the emergence of more virulent recombinants.

## 4. Conclusions and Future Research Needs

Badnaviruses are emerging viruses that cause significant economic loss in yield and quality, especially in the tropical region of the world in crops such as banana, black pepper, citrus, cacao, grapevine, sugarcane, taro, and yam. ICTV listed 68 species in the genus with many hosts (banana, cacao, grapevine, pineapple, sugarcane, sweet potato, taro, and yam) infected by more than one species. In general, viruses in the genus have a restricted host range, infecting a limited number of hosts. The majority of the badnaviruses are transmitted by different species of mealybugs in a semipersistent manner. Wild and uncultivated plants infected with badnaviruses usually remain symptomless or show only mild symptoms. The severity of symptoms of badnavirus-infected cultivated plants generally depends on abiotic factors such as temperature, relative humidity, light intensity, and nutrition of the plants. Hence, more studies are needed on the emerging problems of badnaviruses in the light of changing climatic conditions to identify the factors that trigger symptom development and the severity of the diseases in different hosts. A few studies have also indicated the role of soil pH and nutrients, including micronutrients in symptom development, also need detailed investigation. Similarly, the effect of beneficial and pathogenic organisms including fungal, bacterial, and viral on the badnavirus-infected plants also need to be studied. The information generated from the abovementioned studies would help to develop a strategy to manage the disease.

Currently, the complete genome of many badnaviruses has been determined and many useful assays based on PCR, LAMP, and RPA combined with the lateral flow have been developed for the quick and sensitive detection of viruses that help in the production of virus-free plants. Similarly, methods such as RCA, in situ hybridization, and RT-PCR and IC-PCR are available to differentiate plants infected with episomal and endogenous badnaviruses. The recombinant analysis predicted the occurrence of recombinants among badnaviruses and also suggested the existence of hitherto undetermined diversity in badnaviruses. The intergenic regions of a few badnaviruses have proven that they can be used as promoters for the expression of foreign genes in different hosts. However, the role of different genomic components of the virus on disease development is still unknown.

Methods such as thermo- and chemo-therapies combined with meristem tip culture were reported for the production of virus-free plants of a few badnaviruses. This should be expanded to more hosts, especially the germplasm accessions that are used to breed crops resistant to different pathogens. So far, no varieties resistant to badnaviruses are reported. Hence, more research efforts are needed to develop varieties resistant to badnaviruses using both conventional and biotechnological including gene editing approaches. Recently, endogenous badnaviral sequence-edited banana plants lost their ability to give rise to the episomal form. The role of integrated badnavirus sequences that might impart immunity to their hosts requires further study.

## Figures and Tables

**Table 1:** Details of ICTV-approved species under the genus *Badnavirus*.

Sl. No	Species	Host	Acronym	GenBank Accession Number	No. of ORFs	Genome Length (Nucleotide)
1.	aglaonema bacilliform virus	*Aglaonema modestum*	ABV	MH384837	3	7178
2.	*Badnavirus aucubae* (aucuba ringspot virus)	*Aucuba japonica*	AuRV	LC487411	3	9092
3.	*Badnavirus castaneae* (chestnut mosaic virus)	*Castanea sativa*	ChMD	MT269853	3	7160
4.	banana streak GF virus	Plantain	BSGFV	AY493509	3	7263
5.	banana streak IM virus	*Musa* sp. cv. Mshule	BSIMV	HQ593112	3	7769
6.	banana streak MY virus	*Musa* sp.	BSMYV	AY805074	3	7650
7.	banana streak OL virus	*Musa* sp.	BSOLV	AJ002234	3	7389
8.	banana streak UA virus	*Musa* sp. cv. Likhako	BSUAV	HQ593107	3	7519
9.	banana streak UI virus	*Musa* sp. cv. Kisansa	BAUIV	HQ593108	3	7458
10.	banana streak UL virus	*Musa* sp. cv. Kibuzi	BSULV	HQ593109	3	7401
11.	banana streak UM virus	*Musa* sp. cv. Mbwazirume	BSUMV	HQ593110	3	7532
12.	banana streak VN virus	Plantain	BAVNV	AY750155	3	7801
13.	birch leaf roll-associated virus	*Betula pubescens*	BLRaV	MG686419	4	7864
14.	blackberry virus F	*Rubus* sp.	BVF	KJ413252	3	7663
15.	bougainvillea chlorotic vein banding virus	*Bougainvillea* sp.	BCVBV	EU034539	4	8759
16.	cacao bacilliform Sri Lanka virus	*Theobroma cacao*	CBSLV	MF642736	3	1772
17.	cacao mild mosaic virus	*Theobroma cacao*	CaMMV	KX276640	4	7533
18.	cacao swollen shoot CD virus	*Theobroma cacao*	CSSCDV	JN606110	4	7203
19.	cacao swollen shoot CE virus	*Theobroma cacao*	CSSCEV	MF642720	4	7131
20.	cacao swollen shoot Ghana M virus	*Theobroma cacao*	CSSGMV	MF642724	4	7009
21.	cacao swollen shoot Ghana N virus	*Theobroma cacao*	CSSGNV	MF642725	4	7173
22.	cacao swollen shoot Ghana Q virus	*Theobroma cacao*	CSSGQV	MF642734	4	7102
23.	cacao swollen shoot Togo A virus	*Theobroma cacao*	CSSTAV	AJ781003	9	7297
24.	cacao swollen shoot Togo B virus	*Theobroma cacao*	CSSTBV	L14546	3	7161
25.	cacao yellow vein banding virus	*Theobroma cacao*	CYVBV	KX276641	3	1958
26.	camellia lemon glow virus	*Camellia japonica* cv. Lemon Glow	CLGV	MN542417	3	8203
27.	canna yellow mottle associated virus	*Canna indica* cv. Striped Beauty leaf	CaYMAV	KX066020	3	6966
28.	canna yellow mottle virus	*Alpinia purpurata*	CaYMV	KU168312	3	7120
29.	citrus yellow mosaic virus	*Citrus* *sinensis*	CYMV	AF347695	6	7559
30.	codonopsis vein clearing virus	*Codonopsis lanceolata*	CoVCV	MK044821	3	8112
31.	commelina yellow mottle virus	*Commelina sp.*	ComYMV	X52938	3	7489
32.	cycad leaf necrosis	*Zamia fischeri*	CLNV	EU853709	3	9205
33.	dioscorea bacilliform AL virus	*Dioscorea rotundata*	DBALV	KX008573	3	7609
34.	dioscorea bacilliform AL virus 2	*Dioscorea alata*	DBALV2	KY827395	3	7871
35.	dioscorea bacilliform ES virus	*Dioscorea esculenta*	DBESV	KY827394	3	8106
36.	dioscorea bacilliform RT virus 1	*Dioscorea rotundata*	DBRTV1	KX008574	3	7702
37.	dioscorea bacilliform RT virus 2	*Dioscorea rotundata*	DBRTV2	KX008577	3	7438
38.	*dioscorea* bacilliform RT virus 3	*Dioscorea rotundata*	DBRTV3	MF476845	3	7506
39.	dioscorea bacilliform SN virus	*Dioscorea sansibarensis*	DBSNV	DQ822073	3	7261
40.	dioscorea bacilliform TR virus	*Dioscorea trifida* var. Borelli voucher	DBTRV	KX430257	3	7333
41.	dracaena mottle virus	*Dracaena sanderiana*	DrMV	DQ473478	7	7531
42.	epiphyllum mottle-associated virus	*Hylocereus polyrhizus*	EpMoaV	MH396440	4	7837
43.	fig badnavirus 1	*Ficus carica*	FBV -1	JF411989	3	7140
44.	gooseberry vein banding associated virus	*Ribes rubrum* cv. Holandsky cerveny	GVBAV	JQ316114	3	7659
45.	grapevine badnavirus 1	*Vitis vinifera* var. Ljutun	GBV-1	MF781082	3	7145
46.	grapevine Roditis leaf discoloration-associated virus	*Vitis vinifera*	GRLDaV	HG940503	4	6988
47.	grapevine vein clearing virus	*Vitis vinifera*	GVCV	JF301669	3	7753
48.	green Sichuan pepper vein clearing-associated virus	Zanthoxylum	GSPVCaV	MK371353	3	8070
49.	ivy ringspot-associated virus	*Hedera helix*	IRSaV	MN850490	3	8885
50.	jujube mosaic-associated virus	*Ziziphus jujube*	JuMaV	KX852476	4	7194
51.	kalanchoe top-spotting virus	*Kalanchoe blossfeldiana*	KTSV	AY180137	3	7591
52.	mulberry badnavirus 1	*Morus alba*	MBV-1	LN651258	2	6945
53.	pagoda yellow mosaic associated virus	*Styphnolobium japonicum*	PYMaV	KJ013302	5	7424
54.	pineapple bacilliform CO virus	*Ananas comosus*	PBCOV	GU121676	3	7543
55.	pineapple bacilliform ER virus	*Ananas comosus* var. erectifolius	PBERV	EU377673	3	1510
56.	piper yellow mottle virus	*Piper nigrum*	PYMoV	KC808712	4	7562
57.	polyscias mosaic virus	*Polyscias fruticosa* cv. Ming aralia	PoMV	MH475918	3	7592
58.	rubus yellow net virus	*Rubus* sp.	RYNV	KM078034	6	7836
59.	spiraea yellow leafspot virus	*Spiraea* (Spiraea × bumalda)	SYLSV	MW080369	3	8017
60.	sugarcane bacilliform Guadeloupe A virus	*Saccharum officinarum* var. R570	SCBGAV	FJ824813	3	7444
61.	sugarcane bacilliform Guadeloupe D virus	*Saccharum officinarum* cv.Batavia	SCBGDV	FJ439817	3	7317
62.	sugarcane bacilliform IM virus	*Saccharum officinarum*	SCBIMV	AJ277091	3	7687
63.	sugarcane bacilliform MO virus	*Saccharum officinarum*	SCBMOV	M89923	3	7568
64.	sweet potato pakakuy virus	*Ipomoea batatas*	SPPV	FJ560943	4	8082
65.	taro bacilliform CH virus	*Colocasia esculenta*	TaBCHV	KP710178	6	7641
66.	taro *bacilliform virus*	*Colocasia esculenta*	TaBV	AF357836	3	7458
67.	wisteria badnavirus 1	*Wisteria sinensis*	WBV1	KX168422	4	7326
68.	yacon necrotic mottle virus	*Smallanthus sonchifolius*	YNMoV	KM229702	4	7661

## Data Availability

Not applicable.

## Supplementary Materials

The following supporting information can be downloaded at: https://www.mdpi.com/article/10.3390/pathogens12020245/s1, Table S1: Predicted recombination events detected with badnavirus isolates based on complete genome sequences.
