# Supplementary material for: Emerging and Re-Emerging Diseases Caused by Badnaviruses
Source: Pathogens. 2023 Feb 3;12(2):245. doi: 10.3390/pathogens12020245 (PMC9963457; doi:10.3390/pathogens12020245)
Supplement: Supplementary file 1 [file pathogens-12-00245-s001.zip › pathogens-2149477-supplementary.pdf]

Table S1. Predicted recombination events detected with badnavirus isolates based on complete genome sequences.

Event	Breakpoint		Recombinant	Major parent[a]	Minor parent[a]	Method	*P* value
	Begin	End					
1	3540	2490	EU140339:BSMYV-TRY	Unknown (MW090055:BSMYV-IN6)	AY805074:BSMYV-AUS	**R**GBMCST	6.192×10^{-136}
2	5046	Undetermined (5101)	MW892537:JuMaV-HZ/AKS-6	JN606110:CSSCDV-CI152-09	KX852476:JuMaV-Z6	**R**GMCS	9.606×10^{-44}
3	3174	6270	MW052382:BSMYV-IN9	KF724856:BSMYV-IN3	MW052383:BSMYV-IN10	**R**GBMCST	4.397×10^{-14}
4	6840	Undetermined (6872)	KX852476:JuMaV-Z6	OL739568:JuMaV-HZ/YPH-3	MW116777:PYMoV-Para	R**G**M	2.240×10^{-21}
5	Undetermined (8154)	126	AF357836:TaBV	MW892537:JuMaV-HZ/AKS-6	MG017323:TaBV-Ug75	RG**B**M	5.919×10^{-19}
6	Undetermined (1)	63	MN296502:BSGFV-Yunnan	Unknown (OL423405:BSOLV-5-S68)	FJ824813:SCBGAV1	R**G**C	8.575×10^{-05}
7	Undetermined (3527)	Undetermined (380)	EU140339:BSMYV-TRY	MW090055:BSMYV-IN6	AY805074:BSMYV-AUS	**G**BMCST	3.546×10^{-41}
8	Undetermined (7490)	Undetermined (7526)	HQ852251:GVBAV-RIB9001	MZ220958:GVBAV-13C203	Unknown (MG017323:TaBV-Ug75)	**R**GMT	9.680×10^{-15}
9	3174	6270	MW052382:BSMYV-IN9	Unknown (KF724856:BSMYV-IN3)	MW052383:BSMYV-IN10	RGBMCS**T**	1.004×10^{-19}
10	29	115	AF357836:TaBV	HQ593110:BSUMV-Uganda	MG017321:TaBV-Ke52	**R**GMS	4.415×10^{-13}
11	2808	3003	JN606110:CSSCDV-CI152-09	Unknown (MF642734:CSSGQV-Gha53-15)	MG017327:TaBCHV-Ug10	**R**GMCS	4.645×10^{-13}
12	626	1567	KF724855:BSMYV-IN2	Unknown (MW090055:BSMYV-	KF724854:BSMYV-IN1	RG**B**MCST	4.067×10^{-48}

				IN6)			
13	6658	6786	FJ824813:SCBGAV1	MF642716:CSSTAV-Gha25-15	OL423405:BSOLV-5-S68	**R**GBM	1.119×10^{-23}
14	Undetermined (7127)	Undetermined (68)	ON086739:TaBCHV-PNG-P	MF642720:CSSCEV-GWR198E-13	KP710177:TaBCHV-2	RG**B**M	2.126×10^{-11}
15	Undetermined (18)	122	MG017321:TaBV-Ke52	Unknown (X52938:ComYMV)	AF357836:TaBV	R**G**BMCS	6.082×10^{-17}
16	1	44	MH404156:DBALV2-PNG58_DA	OL874431:BSOLV-ITC1135	MH404163:DBALV2-PNG12_DA	RG**B**M	2.050×10^{-11}
17	7776	Undetermined (8329)	MG686421:BLRaV-BpenGer407526	MZ358192:RYNV-BiH	MG686420:BLRaV-BpubFin407501	G**B**S	6.178×10^{-10}
18	Undetermined (8270)	81	FJ824813:SCBGAV1	Unknown (OL423403:BSOLV-5-S75)	HQ593108:BSUIV-Uganda	R**G**BM	$1.283 X 10^{-05}$
19	3634	3820	MG017324:TaBCHV-Et17	MH404156:DBALV2-PNG58_DA	KX276641:CYVBV-ICS27	R**B**S	8.399×10^{-08}
20	3852	5903	MW052382:BSMYV-IN9	AY805074:BSMYV-AUS	MW052383:BSMYV-IN10	G**B**MCST	7.414×10^{-06}
21	Undetermined (3527)	417	EU140339:BSMYV-TRY	Unknown (KR014107:BSMYV-TO213_2010)	(MW090055:BSMYV-IN6)	**R**GBMCST	1.971×10^{-98}
22	3174	6270	MW052382:BSMYV-IN9	Unknown (KF724856:BSMYV-IN3)	MW052383:BSMYV-IN10	RGBMCS**T**	1.004×10^{-19}
23	8199	92	EU708317:CYMV	MF642725:CSSGNV-Gha63-15	EU708316:CYMV-SOP	R**G**MC	2.785×10^{-22}
24	8188	62	KP710178:TaBCHV-1	MF991952:GRLDaV-VLJ-178	MG017324:TaBCHV-Et17	R**G**M	4.102×10^{-11}
25	8272	120	AF357836:TaBV	OL423403:BSOLV-5-S75)	MG017323:TaBV-Ug75	R**G**M	$2.514.102 \times 10^{-19}$
26	8274	64	HQ593109:BSULV-Uganda	Unknown (OL874431:BSOLV-	MN296502:BSGFV-Yunnan	R**G**M	1.968×10^{-07}

				ITC1135)			
27	4214	4476	MN716771:GVCV-TN1	Unknown (MH475918:PoMV-AR3)	MF476845:DBRTV3	**R**MCS	1.324×10^{-08}
28	1833	2031	ON086738:TaBCHV-PNG-K	Unknown (KM229702:YNMoV-YV1)	MF642724:CSSGMV-Gha57-15	**R**BMCS	7.305×10^{-13}
29	8292	624	JN006806:CYMV-ROL	Unknown (FJ617224:CYMV-Nagri)	JN006805:CYMV-SOJNTU	**R**BMCST	7.075×10^{-10}
30	6097	7245	EU140339:BSMYV-TRY	MW090055:BSMYV-IN6	AY805074:BSMYV-AUS	**G**BMS	3.177×10^{-61}
31	4236	5808	EU140339:BSMYV-TRY	Unknown (MW090055:BSMYV-IN6)	AY805074:BSMYV-AUS	**R**GBMCST	3.079×10^{-127}
32	626	1500	FJ617224:CYMV-Nagri	JN006805:CYMV-SOJNTU	Unknown (JN006806:CYMV-ROL)	R**B**MCST	1.177×10^{-95}
33	Undetermined (8192)-106	106	MG686421:BLRaV-BpenGer407526	Unknown (OL423411:BSOLV-3-S34)	MG686420:BLRaV-BpubFin407501	R**G**MCT	3.838×10^{-20}
34	3174	6270	MW052382:BSMYV-IN9	Unknown (KF724856:BSMYV-IN3)	MW052383:BSMYV-IN10	RGBMCS**T**	1.044×10^{-19}

Recombination events detected using RDP4; [a]The sequence used to infer unknown parent is provided in brackets; Recombinant, major, and minor parents that contributed larger and smaller fractions of recombinant genomes, and the list of methods that detected the event are indicated (R: RDP; G: GENECONV; B: BOOTSCAN; M: MAXCHI; C: CHIMAERA; S: SISCAN; T: 3SEQ) with highest p-value indicated for the method shown in bold.
